# A pregnant female with a large intracranial mass: Reviewing the evidence to obtain management guidelines for intracranial meningiomas during pregnancy

**DOI:** 10.4103/2152-7806.74242

**Published:** 2010-12-25

**Authors:** Ekkehard M. Kasper, Philip E. Hess, Michelle Silasi, Kee-Hak Lim, James Gray, Hasini Reddy, Lauren Gilmore, Burkhard Kasper

**Affiliations:** Department of Neurosurgery, Beth Israel Deaconess Medical Center, One Deaconness Road, Boston, MA 02215, USA; 1 Department of Anesthesiology, Beth Israel Deaconess Medical Center, One Deaconness Road, Boston, MA 02215, USA; 2 Department of Obstetric and Gynecology, Beth Israel Deaconess Medical Center, One Deaconness Road, Boston, MA 02215, USA; 3 Department of Neonatology, Beth Israel Deaconess Medical Center, One Deaconness Road, Boston, MA 02215, USA; 4 Department of Pathology, Beth Israel Deaconess Medical Center, One Deaconness Road, Boston, MA 02215, USA; 5 Department of Neurology/Epilepsy Centre, University of Erlangen, Schwabachanlage 6, 91054 Erlangen, Germany

**Keywords:** Caesarean delivery, meningioma, pregnancy, resection

## Abstract

**Introduction:**

Non-obstetric surgery for intracranial meningioma is uncommon during pregnancy and poses significant risks to both the mother and the fetus. We present a case of a parturient that presented with acute mental status changes and we illustrate the decision making process that resulted in a best-possible outcome.

**Case Description:**

A woman at 29-week gestation presented with acute language and speech deficits and deteriorating mental status after 2 weeks of headache. Imaging demonstrated a large intracranial mass. A multidisciplinary meeting was held to determine the best treatment plan. The decision was to proceed with caesarean delivery under epidural anesthesia to allow intraoperative monitoring of neurological function. Six hours after successful delivery, the patient had acute mental status changes and she was taken to the operating room immediately for resection of her tumor, which turned out to be a clear cell meningioma.

**Discussion:**

Cerebral meningioma is usually a slow-growing tumor; however, during pregnancy, the mass may expand rapidly due to hormonal receptor expression. The presentation of this patient would have normally led to urgent resection of the mass. But the complicating factor was her 29-week pregnancy as standard intraoperative treatment during neurosurgery is known to adversely affect the fetus. A multidisciplinary meeting was critical for this patient’s care, and is recommended by us when treating such patients.

## INTRODUCTION

Non-obstetric surgery during pregnancy is not new, and has been reported for various conditions with a frequency of 0.2–0.79%.[34] It can be performed without increased risk to the fetus.[11] But, brain tumors in a parturient patient are extremely rare scenarios, with an estimated incidence of about seven cases per 125,000 pregnancies.[17,24] They can pose serious challenges to the treating physician balancing the act of treating mother and child during non-obstetric disease.

The overall incidence of intracranial neoplasm is equal if not lower than that in non-pregnant women of childbearing age, and was estimated for meningiomas to be about 1–4.5/100,000 females aged 15–44 years.[20,31] But, for some tumors, and meningiomas in particular, symptoms may flare up due to metabolic changes during pregnancy, causing accelerated growth.[22,23,25,43] A first such observation was published by Bernard in 1898[4] and was related to a case of connective tissue tumor growing rapidly during pregnancy, but it took a few more decades until this specific correlation was recognized for intracranial meningiomas.[12]

There are only a few case reports or small series on the topic available, but the overall lesson learned from these cases is that signs and symptoms can be significantly aggravated antepartum or post-partum and may mimic more common conditions such as hyperemesis gravidarum, eclampsia or puerperal psychosis.[29,30,42] due to either (1) maternal metabolic changes causing fluid retention, vascular engorgement and edema or (2) accelerated tumor growth due to hormonal receptor expression. Triage in such cases needs to be individualized and based on very thorough observation of the clinical setting of such patients.[29]

Previous publications have reported favorable outcomes if managed well.[2,3,16,23,27,28,34] In this particular paper, we illustrate the associated difficulties in decision-making as encountered in a case of a rare, highly vascular,, atypical meningioma variant, the clear cell meningioma. This team effort contributed to the delivery of a healthy premature infant early in the 3^rd^ trimester and subsequent successful removal of the symptomatic intracranial lesion.

### Case illustration

A 40-year-old right-handed Chinese female, 29 weeks pregnant (gravida 3, para 1, 1 miscarriage), presented with headaches and declined mental status. As per the patient’s husband, she complained of constant bitemporal headaches for 2 weeks prior to admission. Word-finding difficulties were noted for 1 week and the patient was unable to finish her sentences. She was able to speak English before the symptoms but developed difficulties even in Chinese, becoming forgetful and at times confused. For 3 days, her symptoms had progressed and she developed nausea and vomiting, becoming very lethargic, spending most of the day sleeping. Past medical history was only relevant for hepatitis-B carrier status and past surgical history was notable for a previous caesarean section for breech.

### Review of Systems

Ultrasound examination had documented an uncomplicated pregnancy, the baby had been moving well and no bleeding or problems with the gestation were noted. Home medications included Telbivudine 600 mg daily, calcium supplement and fish oil; no drug allergies were noted. She lived with her husband and her 12-year-old daughter. There was no use of alcohol, tobacco or drugs and her family history was non-contributory.

### Physical Examination

The patient presented afebrile, with normal vital signs. The general exam was unremarkable. The neurological exam revealed depressed mental status with closed eyes, opening to voice, but non-cooperative. She followed simple commands poorly in English, but better in Chinese. There was an unremarkable cranial nerve exam, with brisk pupillary reactions to light and accommodation.

### Motor

There was normal bulk and tone; no asterixis or myoclonus were noted, but there was right-sided hemiparesis 4/5 throughout with a right-sided pronator-drift.

### Sensory

There was perceived pain and touch; no obvious deficit or paresthesia were noted. Reflexes were symmetric and the plantar response was flexor.

### Labs

Na 139, K 3.3, Cl 108, HCo3 21, BUN 4, Cr 0.4, Gluc 112, Ca 8.1, Mg 1.8, PO4 2.2, ALT 54, AP 53, Tbili 0.5, AST 39, Lip 21, HCG 27344.

WBC 13.4, HCT 32.9, PLT 93.

### Diff

N 84.2, L 11.9, M 3.2, E 0.4, Bas 0.3, PT 12.0, PTT 23.8, INR 1.0.

### Radiographic Work-Up

**Figure 1:**
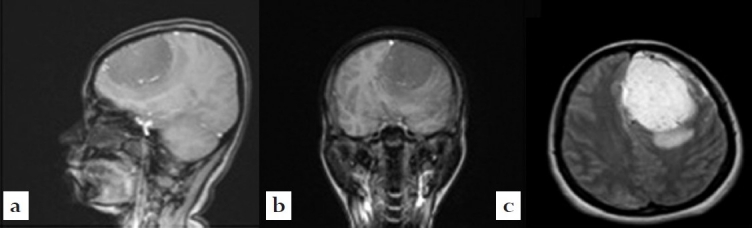
Magnetic resonance imaging of the brain. (a) Sagittal view (T1 weighted); (b) coronal view (T1 weighted); (c) axial view (FLAIR). Imaging demonstrated a 4.9 × 7.2 × 4.8 cm extraaxial mass with surrounding edema and resulting mass effect causing a 14mm rightward shift of the midline structures. There was compression of the frontal horn of the lateral ventricle and the third ventricle, deformity of the midbrain on the left and moderate dilatation of the lateral ventricle. The mass had signal voids which were likely representing vascular flow. There was surrounding vasogenic edema extending throughout the adjacent left frontal white matter.

Because computer tomography (CT) carries a hazardous risk of radiation damage, magnetic resonance imaging (MRI) is the method of choice for this work-up, although it remains suboptimal secondary to the inability to use contrast agents during pregnancy. We obtained a baseline study [Figure 1].

### Hospital Course

The patient was admitted and monitored until imaging could be completed for further assessment. An obstetric consult revealed an uncomplicated pregnancy at 29.2 weeks with unremarkable characteristics by fetal monitoring. The ultrasound revealed breech position. An urgent interdisciplinary conference was called to arrive at a balanced clinical judgement that must weigh a decision to resect the lesion during pregnancy vs. waiting until post-partum if deemed possible.

### Conference

For a comprehensive evaluation and final decision on how to proceed, a multidisciplinary team meeting was called, including Neurosurgery, Maternal-Foetal Medicine, Neonatology and Anaesthesia, to discuss the most appropriate and most safe surgical approach. The management questions presented included the following key points:

From a neurosurgical perspective, a tumor of this size should be removed in a symptomatic patient. Although mainly composed of case reports, the available literature reflects an increased risk to both the mother and the baby over time.

As some tumors do progress rapidly in pregnancy secondary to responses mediated by sex-hormone receptor expression,[7,43] and this patient was demonstrating progressive symptoms, it seemed wise not to wait several weeks (10 weeks to full-term) but to remove the lesion electively as the intrapartum complications can be fatal to the patient and the baby.[29]

Risks to the mother included general endotracheal anesthesia during pregnancy, which is complicated by weight gain, water retention, venous engorgement, upper airway mucosal edema, increased propensity for reflux and aspiration and a decreased functional residual capacity. Furthermore, under general anesthesia, the neurological status can no longer be assessed, which could complicate the evaluation of the patient after emergence. From an anesthesia perspective, the concerns focused around positioning, possible induction of labour, avoiding rapid extremes of blood pressure, which are common during delivery, and also treatment of post-partum hemorrhage during anesthesia in such high-risk pregnancy.[8,17,18]

Particular maternal risk in this scenario however lies in neurological compromise since the tumor may progressively enlarge, cause seizures or a stroke and even herniation. However, the use of diuretics (mannitol or lasix) is not advocated in pregnancy as a sudden decrease in the plasma volume might compromise uteroplacental perfusion and may put the fetus at an unpredictable risk during brain surgery. Conversely, the lack of brain relaxation makes intracranial surgery significantly more difficult. Furthermore, if surgery is needed to be performed, excessive blood loss and subsequent hypotension, hypovolemia and hypoxia are all risk factors for possible harm to the fetus and intrauterine demise.

The set of risks to the fetus included stillbirth, birth defects (more in earlier stages of pregnancy), premature labour, premature delivery and fetal asphyxia. From a fetal perspective, the neonatologist mentioned that the risk for the baby quo ad vitam at this point was about 4–5%, whereas at 31 weeks it would still be 2–3%. Lung maturation had been drug induced with the use of betametasone. For that reason, it was argued that little reduction of fetal risk would be gained by delaying delivery.

Because brain surgery may induce labour, or lead to fetal complications intraoperatively, the consensus was in favor of taking care of the baby first. From an obstetrical perspective, an elective caesarean section, which has a low morbidity/mortality (e.g., blood loss of 500–1000 cc), if performed under epidural anesthesia, could be performed at any time and seemed to be the procedure of choice. Epidural anesthesia would provide the advantage of maintaining stable blood pressure parameters and allowing neurological status assessment during surgery.[1,9] It was indicated that due to a significantly raised intracranial pressure, fluid resuscitation had to be isotonic at all times.

It was decided to proceed with this elective caesarean delivery of the premature fetus as it was felt that an elective premature delivery is preferred over sentinel delivery concurrently with brain surgery. It was assumed that after delivery, the patient could undergo full imaging to complete the pre-operative assessment of the brain and to have the second surgery electively shortly thereafter with significantly reduced risk to the mother.

## FIRST SURGERY

### Caesarean section

After proper informed consent, the patient was taken to the operating room. Epidural anesthesia with mild sedation was induced in the dorsal supine position with slight leftward tilt. A midline vertical incision was then made using her prior caesarean section scar and the dissection was carried out. The peritoneum was entered bluntly. A bladder blade was inserted and a bladder flap was created. The lower uterine segment was found to be underdeveloped and the position of the baby was noted to be transverse. A decision was hence made to perform a classical caesarean section. This was performed without complication and a healthy infant (single, female in transverse position with head to the maternal left side, delivered by vertex) was handed off to the waiting neonatology team, displaying a weight of 1130 g, Apgars of 7 and 8, umbilical cord blood pH of 7.36 and a base excess of 3. The procedure was successfully concluded and the patient was taken to the surgical intensive care unit in an awake and stable condition. The estimated blood loss was <500 cc. Discussion was then held regarding the post-operative care. Given the importance of maintaining euvolemia, it was decided to keep fluids at a minimum to account for the inherent post-caesarean fluid shifts. The plan was to give the patient a 24-h observation period for recovery from her caesarean section before completing her imaging work-up.

**Figure 2:**
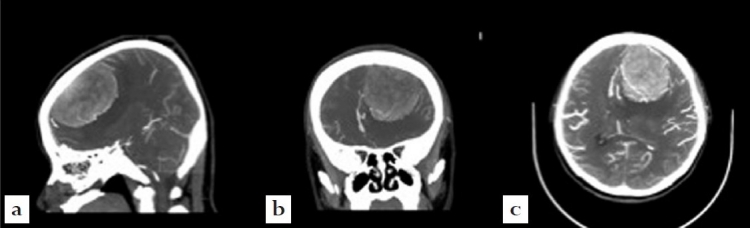
Pre-operative computer tomographic arteriogram of the brain. (a) Sagittal view, (b) coronal view, (c) axial view

The patient was post-operatively stable, with unremarkable labs and vitals for 6 h when she suddenly developed echolalia and progressive aphasia. A STAT computer tomographic arteriogram/computer tomography venogram was performed demonstrating no new hemorrhage or infarct, but progressive swelling and entrapment of the contralateral ventricle. The decision was made to take the patient immediately to the operating room without the possibility of obtaining further MRI [Figure 2].

### Second Surgery

### Left frontal craniotomy for resection of the tumor

The patient underwent general anesthesia and was positioned supine in a Mayfield headrest and she was placed in slight reverse Trendelenburg. She received 100 g of mannitol, 20 mg of Lasix and 10 mg of decadron and was hyperventilated to an arterial partial pressure of carbon dioxide of <30. A wide standard left hemicraniotomy was performed to provide adequate room for dissection. The tumor was easily identified as a spongy hypervascular mass, which was densely adherent to both the dura and the falx. A peritumoral plane was developed under the microscope and the tumor was found to have blood supply from both the anterior cerebral artery and middle cerebral artery as well as the middle meningeal artery. It was carefully dissected without sacrifice of any parenchymal vessels, but under significant blood loss arising from the falx. However, intraoperative vitals were maintained within 10% of the patient’s baseline. Intraoperative fresh frozen pathological analysis revealed a tumor consistent with some type of meningioma.

The case was concluded and the patient was returned to the intensive care unit for further observation.

**Figure 3:**
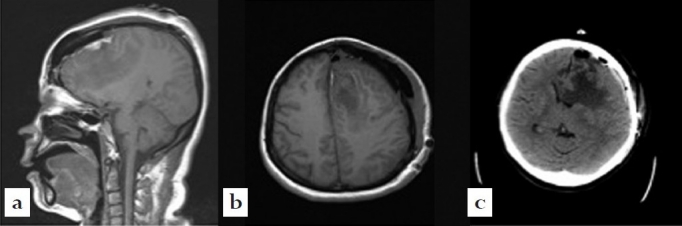
Post-operative imaging of the brain. (a) Sagittal view magnetic resonance imaging (MRI) (T1 weighted), (b) axial view MRI (T1 weighted), (c) axial view computer tomography. Imaging demonstrated gross total resection of the mass

Postoperative imaging (CT/MRI) showed a gross total resection Simpson G2 and no adverse side-effects or complications [Figure 3]. Within 48 h, the patient recovered completely from her pre-operative neurological compromise with a resolved expressive aphasia and no residual weakness.

### Pathology

**Figure 4:**
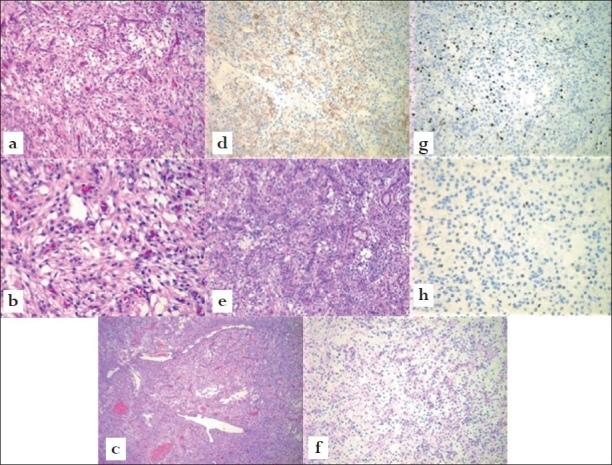
(a) H + E at 10×, (b) 20× showing whorls and sheets of clear cells with oval nuclei and scattered prominent nucleoli, (c) focal “staghorn” blood vessels, H + E at 10×, (d) membranous cellular staining with EMA, 10x, (e) PAS with diastase digestion, (f) intracellular glycogen, 10x, (g) mitotic index by MIB-1 is focally 8–10% and (h) positive nuclear labeling for progesterone.

The lesion was classified as clear cell meningioma, WHO Grade II. Histological sections [Figure 4] showed a moderately cellular, highly vascular tumor. In areas, the blood vessels seemed to show a “staghorn” branching pattern. No necrosis or brain invasion was seen. Although there was a tendency for the cells to form whorls, much of the tumor showed featureless “sheeting.” In syncytial areas, the cell borders were indistinct. Many cells showed cytoplasmic clearing. Periodic acid-Schiff (PAS) with diastase digestion revealed the presence of intracellular glycogen in clear, cell-rich areas. The nuclei were oval and fairly monomorphic, with frequent pseudoinclusions. There were scattered foci with prominent nucleoli. Four mitotic figures were seen in 10 random high-power fields (40×). Tumor cells showed membranous faint labelling with epithelial membrane antigen and were negative for CD34, an endothelial marker that would be positive in hemangiopericytoma. The nuclei were negative for estrogen receptor, but many were positive for progesterone receptor. Mitotic index by MIB-1 was 8–10%.

## DISCUSSION

The management of brain tumors occurring during pregnancy need to be guided by the firm principle of “nihil nocere” (do not do anything that can cause harm) and has to emphasize safety of both the mother and the fetus as the primary goal of treatment. Some patients can be managed conservatively antepartum, but urgent intervention may be required in cases of (1) malignancies, (2) hydrocephalus and (3) relatively benign neoplasm that shows growth and progressive signs and symptoms of mass effect leading to neurological deficit and even incipient herniation, as in our case presented here.

Meningiomas are a rather frequently encountered neoplasm in neurosurgical practice. These tumors arise from the arachnoid cells and account for 10–20% of the brain tumors seen in a general neurosurgical oncology population and may also rarely occur extradurally or even extracranially.[35,37] Meningiomas variably express hormone receptors for progesterone, androgen, estrogen and placenta growth factor,[7,14,15,30,33,38,39,44,45,47] as well as exogenous hormones,[10] and their response to increased serum progesterone levels during the second half of pregnancy may account for accelerated growth.[5,19,21,40,44] This explains the sudden presentation as a neurosurgical emergency in some circumstances.[13,36,49] However, not all meningiomas are equal and data from the literature indicates that the clear cell meningioma encountered in our patient is exceedingly rare.[3,6,26,32] Positive sex hormone receptor status for progesterone in conjunction with an increased MIB proliferation index reflects a prognosis for the patient with increased recurrence rates.[39,41]

We thus may have to treat this patient in the early post-operative setting with adjuvant external beam radiation therapy of the involved field.

During the third trimester, up-front delivery of a viable fetus is the first choice to keep the risk of maternal death not higher than in non-pregnant females undergoing such surgery.[46,48]

In our case, the interdisciplinary management of this scenario proved to be exceptionally suited to manage our maternal patient well, and the premature child was found to be healthy at delivery and showed normal development at the time of writing.
